# Predictive correlations and mechanical assessment of asphalt mixtures modified by sasobit redux and crumb rubber

**DOI:** 10.1038/s41598-026-66628-5

**Published:** 2026-08-24

**Authors:** Mokhtar F. Ibrahim, Ahmed M. Sawan, Metwally G. Al-Taher, Mahmoud El-saied Ali Solyman, Mohamed Ibrahim El-Sharkawi Attia

**Affiliations:** 1 Civil Engineering Department, Faculty of Engineering, Suez University, Suez, Egypt; 2 Civil Engineering Department, Faculty of Engineering, Arish Branch, Sinai University, North Sinai, Arish, Egypt; 3 Construction Engineering & Utilities Department, Faculty of Engineering, Zagazig University, Zagazig, Egypt

**Keywords:** Sasobit Redux, Crumb Rubber, WMA, HMA, Dynamic Modulus, Flow Number, Traditional Asphalt Tests, Shear Triaxial Resistance, Engineering, Environmental sciences, Materials science

## Abstract

To satisfy advanced pavement design methodologies such as the Mechanistic-Empirical Pavement Design Guide (MEPDG), accurate characterization of asphalt mixtures through parameters like the dynamic modulus│E*│is critical. However, obtaining these parameters via advanced laboratory testing demands specialized equipment and complex configurations. This study addresses this limitation by developing empirical correlations to predict│E*│and shear triaxial resistance (STR) directly from conventional, highly accessible laboratory tests, specifically Marshall, unconfined direct compression, indirect tensile strength (ITS), and wheel tracking tests. The experimental program evaluated both Hot Mix Asphalt (HMA) as control mixes and Warm Mix Asphalt (WMA) prepared with diverse aggregate gradations. To enhance the mechanical and environmental performance of the mixtures, two additives were evaluated, Sasobit Redux (SR), blended into the bitumen utilizing the wet process at dosages of 1.0% to 5.0% by weight of bitumen; and Crumb Rubber (CR), incorporated into the total mix weight via the dry process at dosages of 0.5, 1.0, 1.5, 2.5, and 5%. Specimen testing was conducted across a comprehensive matrix of loading rates and environmental conditions. The study successfully established highly predictive empirical equations as primary outcomes. The experimental data revealed that incorporating SR and CR additives significantly altered and enhanced key mechanical properties, specifically enhancing Marshall stability, rutting resistance, unconfined compressive strength, and ITS. Quantitatively, the statistical analysis proved the strong predictive capability of conventional tests, yielding exceptional mathematical correlations within this specific dataset with an R² value of 0.9831 for the│E*│versus rut depth (RD) relationship and an R² value of 0.8751 for the│E*│versus STR relationship. Consequently, these newly developed empirical equations deliver a validated, cost-effective framework for accurately estimating complex pavement design inputs directly from routine laboratory testing data.

## Introduction

The service life of an asphalt surface course is evaluated through its fatigue and permanent deformation characteristics under cyclic traffic loading^1^. Consequently, enhancing asphalt mixture properties, specifically rutting resistance and moisture susceptibility, extends pavement service life, thereby promoting infrastructural sustainability^2^. HMA is a combination of two primary ingredients: aggregates and asphalt binder^3^. To provide an alternative to traditional Hot Mix Asphalt (HMA), modified asphalt utilizes binder additives that enhance aggregate adhesion, mixture performance, and workability^4^. Incorporating additives extends pavement service life and retards deterioration. These modifiers are introduced either into the bitumen via the wet process or directly into the mixture during plant production using the dry process^5,6^. To address these critical performance deficiencies, the incorporation of chemical, polymer, and sustainable waste additives into asphalt mixtures has emerged as a highly effective and widely adopted strategy. Additives such as synthetic waxes (e.g., Sasobit), chemical modifiers, and recycled materials like crumb rubber (CR) alter the fundamental rheological behavior of the bituminous binder and significantly improve the aggregate-binder interfacial bond. In HMA, these modifiers function through dual mechanical mechanisms. At elevated temperatures, they increase the complex shear modulus G* and the elasticity of the binder, restricting shear flow and mitigating rut depth under heavy channelized wheel loads. Numerous investigations have evaluated the performance of pavements modified with recycled materials. These include reclaimed asphalt pavement (RAP), glass, steel slag, crumb rubber (CR), polymers, and waste oils such as waste frying oil (WFO) and waste engine oil (WEO)^7–10^.

Implementing Warm Mix Asphalt (WMA) technologies reduces production temperatures by up to 40 °C, offering a direct mechanism for cutting greenhouse gas emissions and energy consumption during mixture fabrication. However, the lower production temperatures associated with WMA can compromise early-stage stiffness and increase the pavement’s susceptibility to moisture damage and high-temperature rutting. To mitigate these performance deficits, researchers have extensively investigated the incorporation of organic, chemical, and recycled polymeric additives to modify the viscoelastic behavior of the asphalt matrix. Among organic WMA modifiers, Fischer-Tropsch synthetic waxes, such as Sasobit and its advanced formulation Sasobit Redux, are widely utilized. These organic additives function via a temperature-dependent phase change mechanism: they completely dissolve in the bitumen at temperatures above their melting point (typically > 85 °C to 100 °C), executing a marked reduction in binder viscosity that facilitates effective aggregate coating and compaction at lower temperatures^11–13^. Utilizing scrap tires in asphalt pavements represents an economically viable and ecologically sustainable strategy for waste management^14,15^. Crumb rubber is integrated into asphalt pavements utilizing two primary techniques: the wet and dry processes^16,17^. While recent research focuses on reducing production and compaction temperatures to improve workability, the lack of a standardized Warm Mix Asphalt (WMA) design framework highlights the need to understand how these additives interact within the mixture.

Numerous studies have sought to establish correlations between various asphalt mixture parameters. For instance, Rondon et al. (2018) investigated the relationship between mathematically derived dynamic modulus and laboratory-measured resilient modulus^18^. Beyond traditional empirical modeling, recent literature has deployed machine-learning algorithms to predict the physical and engineering characteristics of asphalt mixtures^19,20^, utilizing soft computing techniques such as artificial neural networks (ANNs), genetic algorithms (GAs), and fuzzy logic^21^. Specifically, researchers have successfully utilized standard Marshall mix design inputs, including air voids, density, and voids in mineral aggregate (VMA), to predict key volumetric and mechanical outcomes like Marshall stability (MS), Marshall flow (MF), and the Marshall quotient (MQ)^22^.

Hence, this paper evaluates the production of Warm Mix Asphalt (WMA) incorporating two modifier incorporation methods: the wet process utilizing Sasobit redux (SR) blended with bitumen, and the dry process utilizing crumb rubber (CR) introduced directly into the aggregate mixture. The performance of these modified WMA mixes is benchmarked against conventional Hot Mix Asphalt (HMA). Additionally, this study aims to establish correlations among various asphalt mixture parameters to predict non-conventional performance indicators. Finally, various experimental parameters, including Marshall stability, direct compression, indirect tensile strength (ITS), and rut depth obtained from different mix types and aggregate gradations, were utilized to develop predictive correlations. Furthermore, these conventional parameters served as key inputs to predict advanced performance indicators, specifically the dynamic modulus E* and shear triaxial resistance (STR).

## Research methodology

This investigation represents an extension of the foundational research previously established by Al-Taher et al. (2024)^23^. In their previous study, Sasobit Redux (SR) was comprehensively evaluated as a Warm Mix Asphalt (WMA) additive across a dosage spectrum of 0%, 1.0%, 1.5%, 2.0%,2.5%, 3.0%, 4.0%, and 5.0% by weight of the bitumen. The empirical findings demonstrated that an optimum concentration of 2.5% SR successfully mitigated the high temperature demands of conventional Hot Mix Asphalt (HMA), effectively lowering the mixing and compaction temperatures to 117 °C and 111 °C, respectively. Concurrently, this optimum formulation yielded substantial performance enhancements, including an increase in Marshall stability by up to 25%, a reduction in rut depth by up to 28%, and a marked improvement in moisture susceptibility resistance. To significantly advance these baseline findings, the current study expands the research horizon through a multi-faceted methodology characterized by four primary advancements.

- First, to evaluate a hybrid asphalt modification by combining crumb rubber (dry process) with SR matrix (wet process) to enhance the workability and the engineering properties of the asphalt mixtures.
- Second, to evaluate the viscoelastic behavior and internal friction of the modified mixtures using advanced complex modulus │E*│and shear triaxial resistance (STR) testing.
- Third, to establish mathematical correlations to predict the complex modulus│E*│and shear triaxial resistance (STR) from conventional tests.
- Fourth, to develop predictive mathematical models using multiple linear regression in SPSS to quantify the effects of independent modifiers on pavement performance.

Finally, it should be noted that all samples containing crumb rubber, as well as the triaxial shear test, the dynamic modulus of elasticity│E*│ test, and the flow number (FN) test, pertain to the current study, while the remaining samples and tests were compiled from previous work. Also, The dry process was deliberately selected in this study to preserve the low mixing and compaction temperature benefits achieved by the pre-optimized Sasobit Redux (SR) Warm Mix Asphalt (WMA) matrix, prevent excessive binder viscosity and phase separation that would occur from compounding two concurrent wet-process modifications, and enhance structural shear and rutting resistance by utilizing the rubber particles as an elastic, interlocking aggregate skeleton, thereby providing a highly practical, cost-effective framework easily implemented by standard production plants.

## Research objectives

The study objectives are summarized as follows:

- To evaluate the effect of a combination of dry-processed crumb rubber (CR) from waste tires into an optimized asphalt mix containing Sasobit redux (SR) by conducting traditional and advanced tests.
- To advance conventional empirical testing by conducting complex modulus│E*│and shear triaxial resistance (STR) testing, thereby capturing the true viscoelastic behavior, internal friction, and cohesion of the modified mixtures under complex loading frequencies and stress states.
- To predict and establish mathematical relationships that correlate conventional test outcomes with advanced, high-cost experimental data, specifically complex modulus │E*│and shear triaxial results, provided that these empirical models are defined strictly as study-specific correlations, valid solely for the tested materials, variables, and testing conditions utilized in this investigation.

## Materials

**Fig. 1:**
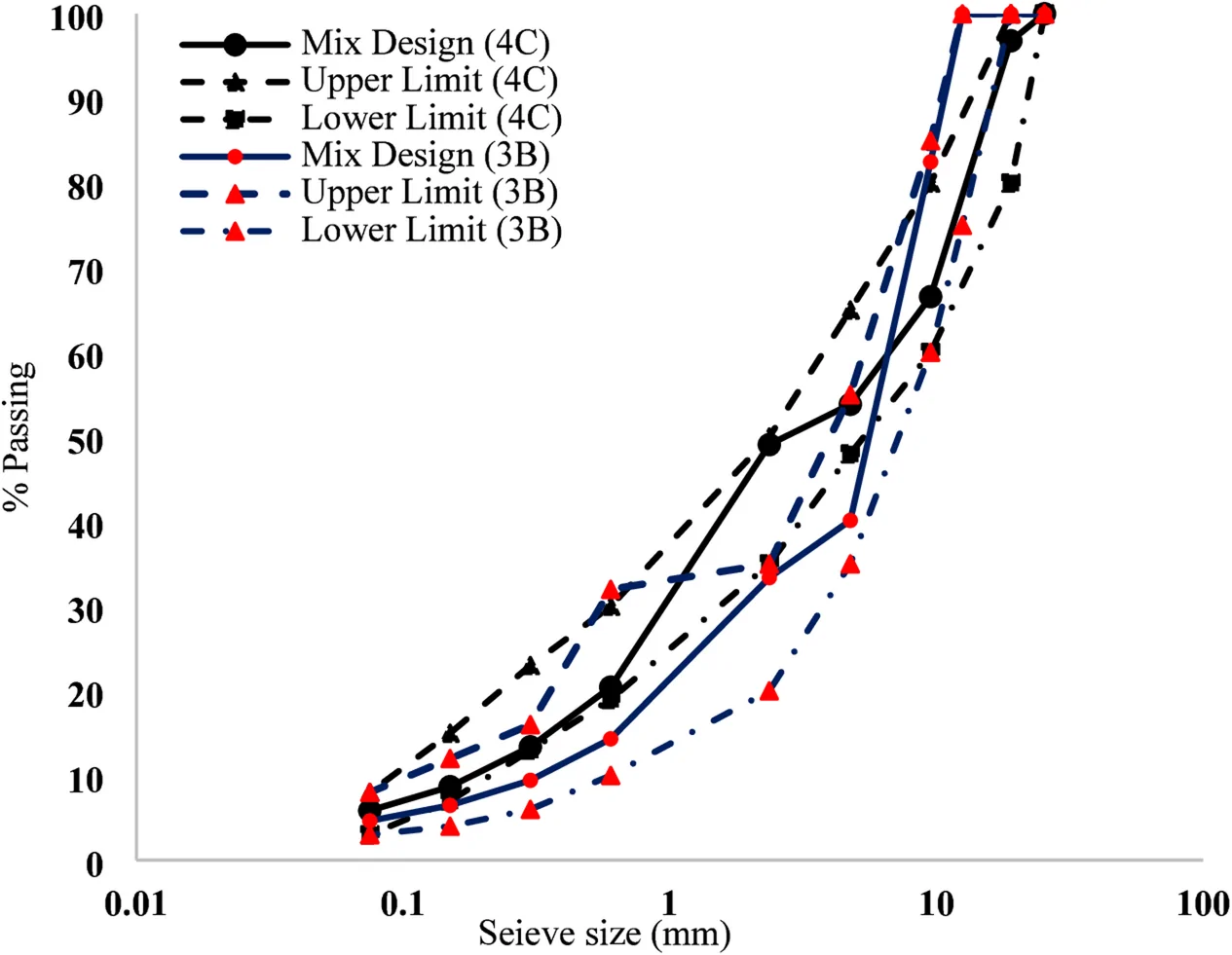
Aggregate Gradation Curves, 4 C and 3B.

**Fig. 2:**
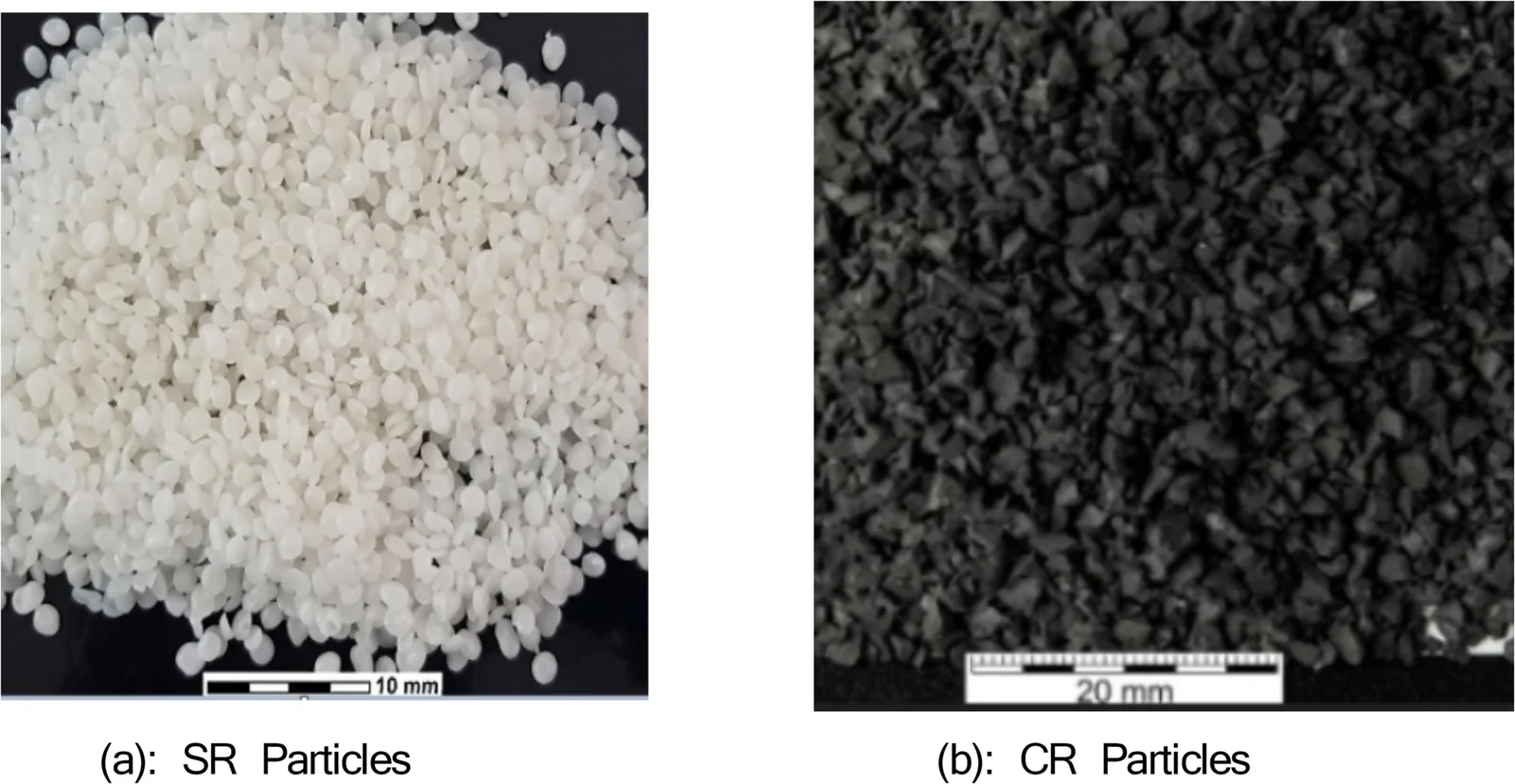
Shape of Sasobit (SR) and Crumb Rubber (CR).

This study utilized Suez asphalt binder (AC-60/70) penetration grade for the preparation of all examined asphalt mixtures. The physical characteristics of the utilized asphalt are presented in Table 1. One aggregate source and two gradations were used. The coarse aggregate consisted of crushed dolomite stone. The fine aggregate employed was siliceous crushed dolomite sand. The mineral filler consisted of cementing dust with a bulk specific gravity of 2.90. Table 2 presents the physical properties of the aggregate. Figure 1 illustrates the gradation curves for the two gradations (dense-graded 4 C and coarse-graded 3B) for wearing surface layers in accordance with the Egyptian specifications standards^24^. Sasobit Redux (SR) was utilized in this study as a warm-mix asphalt additive. This substance comprises synthetic Fischer-Tropsch (FT) wax and other petroleum-derived waxes, with a congealing point ranging from 72 °C to 83 °C. It is a synthetic aliphatic hydrocarbon wax and a derivative additive of coal. SR is entirely soluble in bitumen and markedly decreases viscosity. In the cooling phase, SR initiates crystallization at 60 °C, hence expanding the compaction window^24^. Table 3 delineates the characteristics of SR, while Figure 2a illustrates its shape. Crumb Rubber (CR) was utilized in this study as a component of fine materials in a dry process. The gradation and attributes of CR are presented in Tables 4 and 5, respectively, and its shape is illustrated in Figure 2b.

**Table 1:** Physical properties of asphalt binder.

Property	Designation No.	Result	Specification Limits [24]
Penetration at 25 °C, 0.1 mm	ASTM D5	63	(60–70)
Kinematic Viscosity at 135 °C, Cst	ASTM D2170	469	≥ 320
Rotational Viscosity at 135 °C, CP	ASTM D4402	722	≤ 3000
Flash Point, °C	ASTM D93	270	≥ 250
Softening Point, °C	ASTM D36	49	(45–55)

**Table 2:** Physical Properties of Aggregate.

Property	ASTM Designation No.	Coarse Aggregate Size (1)	Coarse Aggregate Size (2)	Crushed Sand	Natural Sand	Specification Limits [24]
Bulk (S.G)	ASTM C127 & ASTM C128	2.583	2.600	2.752	2.648	-------
Saturated and dry surface (S.G)		2.645	2.652	--------	-------	-------
Apparent (S.G)		2.752	2.745	--------	-------	-------
Los Angeles abrasion - after 100 revolutions; % - after 500 revolutions; %	ASTM C131 & ASTM C535	7.30% 22.40%	8.45% 23.60%			≤ 10% ≤ 40%
% Water absorption	ASTM C127 & ASTM C128	2.20%	2.35%	3.60%	1.60%	≤ 5%
% Decomposed in the water	ASTM C127 & ASTM C128	0.25%	0.22%			≤ 1%
Atterberg limits: - Plastic Limit; % -Liquid Limit; %	ASTM D4318	----	----	No- plasticity	No- plasticity	No- plasticity

**Table 3:** Properties of the Sasobit Redux (SR)^25^.

Properties		Units	Specification	Typical Values	Values and Ranges
Quantitative	Congealing Temperature	^o^C	ASTM D 938	78.00	72–83
	Penetration at 25 C	0.10 mm	ASTM D 1321	23.00	16–30
	PH			Neutral	NR
	Density	Kg/m^3^		580.00	NR
	Molecular Mass	Dalton		620.00	NR
	Distribution of Carbon			1.30%	NR
	Content of Oil			0.90%	NR
Qualitative	Odor				No odor
	Visual Color				White
	Physical State				Granules
	Components				Alpha Tee hydrocarbon
	Solubility in water				Insoluble

NR: No Range.

**Table 4:** Gradation of Crumb Rubber (CR).

Sieve Size		% Passing
inch	mm	Design
No.4	4.75	100.00
No.8	2.36	37.75
No.16	1.18	15.25
No.30	0.60	7.75
No.50	0.30	3.25
No.100	0.15	1.25
No.200	0.075	0.00

**Table 5:** Properties of CR.

Properties	
Molecular Formula	C_5_H_8_
Specific gravity	1.15
Melting point (^o^ C)	170 °C
Physical state	Clear black powder
Young Modulus (E)	2600–2900 MPa
Tensile Strength (σ)	40–70 MPa
Elongation at break	25–50%

## Modified bitumen and modified asphalt mixtures preparation

Modified binders were prepared as explained in the previous work^23^. The mixing process reportedly does not influence the characteristics of the resulting Sasobit-modified asphalt binder^26,27^. Dry process procedures for crumb rubber were employed in the manufacture of asphalt mixtures. The aggregate was subjected to heating in an oven at the design temperature. The CR, expressed as a percentage of the total weight of the combination, was used at percentages of 0.50, 1.0, 1.5, 2.5, and 5%, thereafter combined with either the unmodified or modified binder, and then manually mixed to achieve a homogeneous mixture. The aggregate, CR, and bitumen treated with SR were simultaneously combined. The aggregate and asphalt binder were utilized to create asphalt mixtures with gradations 4 C and 3B. The Marshall design was executed in accordance with ASTM D 6927^28^for different mixtures under varying conditions to ascertain their optimal asphalt contents and associated Marshall properties at these asphalt percentages. The mixing process reportedly does not influence the characteristics of the resulting Sasobit-modified asphalt binder^29^. Several tests are necessary to ascertain the initial strength (initial durability indicators) of the formulated mixtures. The tests encompassed the Wheel Tracking Test, Triaxial Shear Test, Indirect Tensile Test, Unconfined Compression Test, Dynamic Modulus, Flow Number, and Marshall Stability-Flow Test.

## Testing

A variety of experiments were necessary to ascertain the engineering and mechanical qualities of the analyzed mixes. The tests encompass primary qualification assessments for bitumen and aggregates, as detailed in Table 6, concerning engineering properties. The mechanical properties of the control and modified mixes were assessed by a range of conventional tests, including Marshall, Indirect Tensile Strength (ITS), Unconfined Compression (UC), and wheel tracking tests. Subsequently, informed by the optimal outcomes from prior assessments, advanced evaluations were performed, including triaxial shear tests and dynamic modulus testing. Three specimens were tested for each mixture and conditioning case.

**Table 6:** Qualification conducted tests and their specification.

No.	Test	ASTM Specifications
1	Particle gradation for coarse and fine aggregates	C136
2	Aggregate specific gravities test	C127
3	Los Angeles test	C131
4	Water abrasion and disintegration test	C127
5	Atterberg limits tests	D4318
6	Gradation for mineral filler	D546
7	Asphalt penetration test	D5
8	Asphalt softening point test	D36
9	Asphalt flash point test	D93

Marshall test was conducted in accordance with ASTM D 6927^28^. The Marshall Quotient (MQ) was computed using Eq. (1), with an acceptable range of 200–500 kg/mm as per the specifications of the Ministry of Road Transport and Highways(2013)^30^. The testing temperature was 60 °C.

1 $$\:\mathrm{M}\mathrm{Q}\:=\:\frac{\mathrm{M}\mathrm{a}\mathrm{r}\mathrm{s}\mathrm{h}\mathrm{a}\mathrm{l}\mathrm{l}\:\mathrm{S}\mathrm{t}\mathrm{a}\mathrm{b}\mathrm{i}\mathrm{l}\mathrm{i}\mathrm{t}\mathrm{y}}{\mathrm{M}\mathrm{a}\mathrm{r}\mathrm{s}\mathrm{h}\mathrm{a}\mathrm{l}\mathrm{l}\:\mathrm{F}\mathrm{l}\mathrm{o}\mathrm{w}\:}\:[\mathrm{k}\mathrm{g}/\mathrm{m}\mathrm{m}]$$

Following the determination of the Optimum Asphalt Content (OAC) values, samples were prepared for the indirect tensile strength (ITS) test in accordance with ASTM D6931^31^. The ITS test is closely associated with the cracking characteristics of the pavement. The ITS value was determined using Eq. (2)^31^. The testing temperature was 25 °C.

2 $$\:\mathrm{I}\mathrm{T}\mathrm{S}=\left(\frac{2P}{{\uppi\:}\mathrm{*}\mathrm{H}\mathrm{*}\mathrm{D}}\right),\:[\mathrm{k}\mathrm{g}/\mathrm{c}\mathrm{m}2]$$

The compressive strength value (CS) in the direct compression test was computed by using Eq. (3) in kg/cm^2^ according to ASTM D1074^32^. The test was carried out at a temperature of 25 °C.

3 $$\:\mathrm{D}\mathrm{C}=\frac{4\mathrm{P}}{{\uppi\:}{\mathrm{D}}^{2}},\:[\mathrm{k}\mathrm{g}/\mathrm{c}\mathrm{m}2]$$

Where P is the failure load (Kg), H is the specimen height (cm), and D is the specimen diameter (cm).

The wheel tracking test assesses the rate of permanent deformation caused by a moving focused load in accordance with Egyptian criteria^24^. The test measured the rate of permanent deformation caused by a dynamic wheel load and was utilized to assess the susceptibility of asphalt mixtures to premature failure due to deficiencies in the aggregate structure, insufficient binder stiffness, or moisture degradation. This assessment evaluates the rut depth and the frequency of passes or the duration until the final rut depth is achieved. The sample was created by initially combining the ingredients at the designated mixing temperature, subsequently compacting the mixtures using a 53 kg steel roller for 14 cycles, and finally allowing the specimen to cool for 24 h. Samples were fabricated with dimensions of 305 × 305 × 50 mm. The specimens underwent testing at 60 °C for a duration of 1 h^24^.

The triaxial shear test for asphalt mixtures typically includes the shear strength of the specimen under different loading conditions. Shear strength is typically expressed as the peak shear strength, which is the maximum shear stress that the specimen can withstand before failure. These parameters can provide valuable information on the mechanical behavior of the asphalt mixture and can be used to evaluate the performance of the mixture under different loading conditions. The cylindrical specimen used in the triaxial shear test for asphalt mixtures is typically prepared by compacting a sample of the asphalt mixture into a cylindrical mold with a diameter of 4 inches (101.6 mm) and a height of 4 inches (101.6 mm). To prepare the specimen, the asphalt mixture was first heated to a temperature that allows for proper compaction (about 135 °C). At each confining pressure, only one specimen was proven enough for high rates of loading (50 mm/ min) with an elevated temperature of 60 °C. These values of compressive strength for each mix were outlined in the Mohr diagram to assess its shear strength parameters (C&Ф). In this study, the cohesion (C), internal friction angle (ϕ), and shear strength of mixtures were calculated by an unconfined compressive strength test. According to the Coulomb-Mohr strength theory, C and ϕ can be solved by the formula. Then, the shearing triaxial resistance (STR) of each mix was estimated using Eq. (4).

4 $$\:STR=\frac{4C}{1-{sin}\varPhi\:}*\sqrt{\frac{1+{sin}\varPhi\:}{\mathrm{1-sin}{\Phi\:}}}$$

**Fig. 3:**
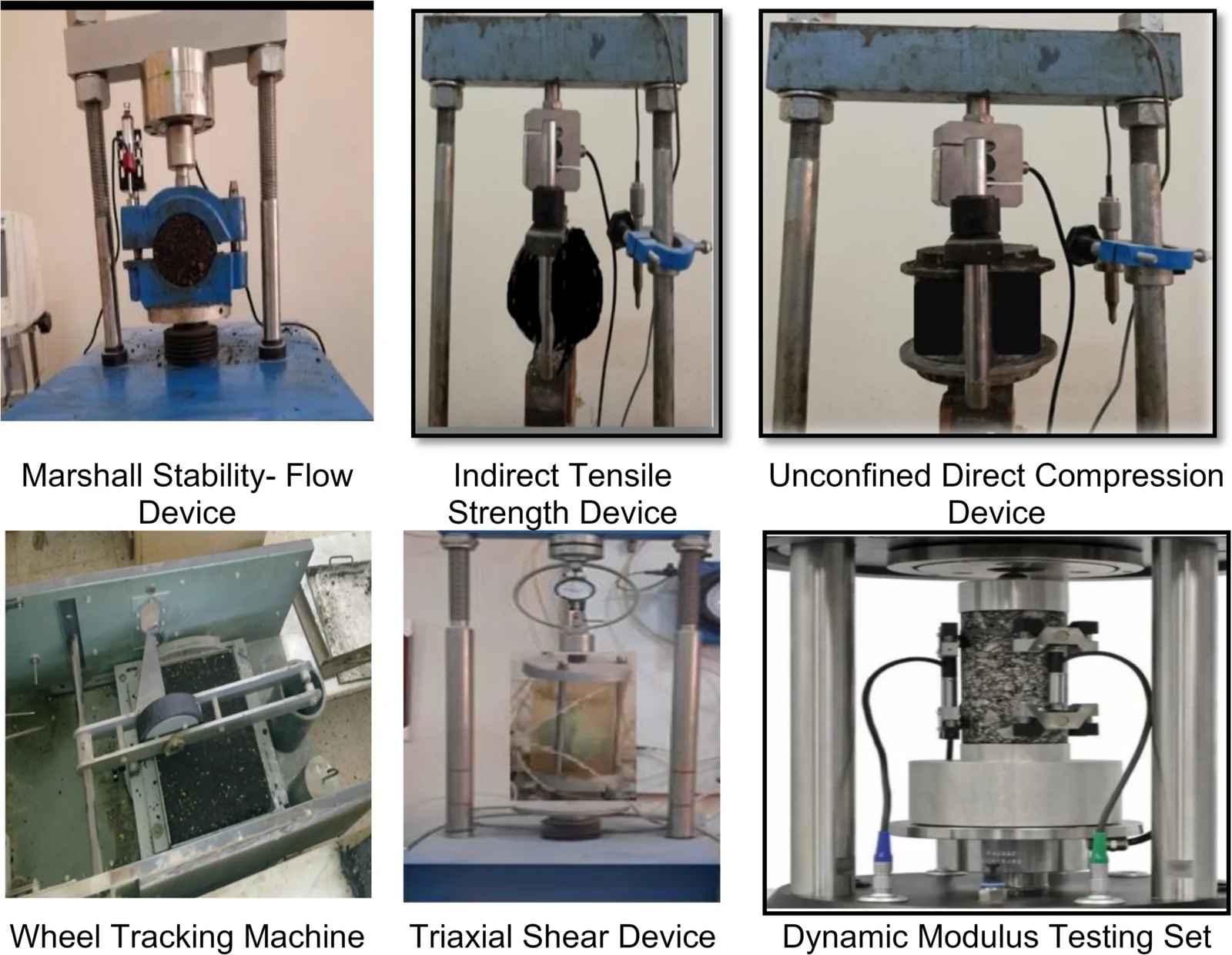
Photographic documentation of the testing apparatus and specimens.

The dynamic modulus │E*│ is an essential indicator to evaluate the performance of asphalt mixtures over varying temperatures and frequencies. The examination was conducted in accordance with AASHTO TP62. The dynamic modulus │E*│ is a measure utilized to assess rutting and fatigue cracking distress forecasts in the Mechanistic-Empirical Pavement Design Guide (MEPDG). Numerous factors influence dynamic modulus values, including aggregate gradation, air void content, binder shear dynamic modulus G*, and binder viscosity. Consequently, this study conducted tests on both traditional and modified mixes. The dynamic modulus test was applied at six different frequencies (0.1 Hz, 0.5 Hz, 1 Hz, 5 Hz, 10 Hz, and 25 Hz). At each frequency, results were recorded for four different temperatures (4.4 °C, 21.1 °C, 37.8 °C, and 54.4 °C). The flow number (FN) test was performed at 54.4 °C under repeated compressive haversine loading (one cycle with 0.1 s loading time and 0.9 s resting time) to quantify the vertically accumulated permanent strains as a function of loading cycles following the │E*│ testing. The test assesses rutting resistance; hence, it was conducted on both control and modified mixtures. Figure 3 shows photographic documentation of the testing apparatus and specimens.

## Results and discussions

### Marshall test results

Tables 7 and 8 present the Marshall characteristics for previous and current studies (HMA and WMA mixes), which explain the effect of using SR and CR on the Marshall Test results. The results presented all parameters, optimum asphalt content (OAC), air voids (AV%), Marshall Stability and flow, voids in mineral aggregates (VMA%), voids filled with bitumen (VFB%), and Marshall Quotient (MQ) for the two gradations 4 C and 3B. All results met the required specification according to Egyptian standards^17^. The findings indicated that the OAC% values were nearly identical across all combinations, ranging from 5.13 to 5.92%. The stability of the gradations 4 C and 3B. Marshall Stability values were observed to rise with higher CR and SR percentages for the two gradations. Gradation 4 C had the best stability value (1.0% CR and 2%SR) by 39%, whereas gradation 3B had the maximum stability (2.5% CR and 2%SR) by 33%. Raising the proportion of CR and SR did not result in a substantial increase in flow, and the improvement was unclear for two gradations. The Marshall Quotient (MQ) at various percentages for CR and SR in gradations 4 C and 3B. MQ at (1.0% and 2.5%) CR with 2%SR for gradations 4 C and 3B, respectively, within the limit specified in the Ministry of Road Transport and Highways 2013 Specification^30^. To satisfy the structural and environmental durability requirements of pavement design, all parameters were evaluated against the threshold limits mandated by the Egyptian standards.

**Table 7:** Results of marshall test for control and modified mixes for the two gradations, 4 C and 3B, investigated by the previous work^23^.

Mix Type	Mix Gradation	Marshall Properties							
		OAC (%)	Stability (kg)	Flow (mm)	Unit Weight (gm/cm^3^)	AV (%)	VMA (%)	VFB (%)	MQ (kg/mm)
CM (without additives)	4 C	5.58	1214	3.40	2.364	3.80	15.95	76.00	357
	3B	5.40	964	3.68	2.338	3.90	15.55	74.50	262
WMA (1.0%SR)	4 C	5.86	1236	3.43	2.350	4.00	16.65	76.00	360
	3B	5.36	1055	3.79	2.347	3.50	15.20	77.00	278
WMA (1.5%SR)	4 C	5.78	1291	3.33	2.347	4.25	16.70	74.50	388
	3B	5.53	1173	3.12	2.330	4.10	15.90	74.00	376
WMA (2.0%SR)	4 C	5.86	1364	3.30	2.345	4.20	16.80	75.00	413
	3B	5.33	1205	3.05	2.335	4.20	15.60	72.50	395
WMA (2.5%SR)	4 C	5.92	1509	3.23	2.355	3.80	16.45	77.00	467
	3B	5.46	1309	2.52	2.343	3.70	15.45	76.50	519
WMA (3.0%SR)	4 C	5.65	1255	3.28	2.361	3.80	16.10	76.00	383
	3B	5.33	1027	3.23	2.335	4.20	15.60	73.00	318
WMA (4.0%SR)	4 C	5.87	1082	3.66	2.352	3.90	16.60	76.50	296
	3B	5.38	991	3.43	2.332	4.20	15.75	73.50	289
WMA (5.0%SR)	4 C	6.00	982	3.94	2.340	4.30	17.10	75.00	249
	3B	5.47	891	3.53	2.338	3.80	15.70	76.00	252

**Table 8:** Results of Marshall Test for Control and Modified Mixes for the two Gradations, 4 C and 3B, investigated by the current work.

Mix Type	Mix Gradation	Marshall Properties							
		OAC (%)	Stability (kg)	Flow (mm)	Unit Weight (gm/cm^3^)	AV (%)	VMA (%)	VFB (%)	MQ (kg/mm)
0.5%CR	4 C	5.83	1168	3.96	2.332	4.30	16.65	74.50	295
0.5%CR + 1.5%SR		5.73	1177	3.68	2.335	4.25	16.50	74.50	320
0.5%CR + 2.0%SR		5.40	1255	3.38	2.347	4.20	15.85	73.50	371
0.5%CR + 2.5%SR		5.80	1209	3.43	2.335	4.10	16.60	75.00	352
0.5%CR + 3.0%SR		5.58	1109	3.56	2.343	4.10	16.15	75.00	312
1.0% CR		5.70	1218	3.56	2.320	4.30	16.45	74.50	342
CM + 1.0%CR + 1.5%SR		5.57	1305	3.40	2.330	4.00	16.00	75.00	384
CM + 1.0%CR + 2.0%SR		5.92	1682	3.43	2.317	4.20	16.70	75.00	490
CM + 1.0%CR + 2.5%SR		5.73	1418	3.81	2.340	3.40	15.65	78.50	372
CM + 1.0%CR + 3.0%SR		5.50	1273	3.63	2.331	4.10	15.95	74.50	351
CM + 1.5%CR		5.13	1250	2.69	2.355	3.70	15.75	77.00	465
CM + 1.5%CR + 1.5%SR		5.70	1309	3.78	2.322	3.80	15.90	76.50	346
CM + 1.5%CR + 2.0%SR		5.70	1436	3.89	2.330	3.40	15.60	78.50	369
CM + 1.5%CR + 2.5%SR		5.57	1318	3.35	2.328	3.60	15.60	77.00	393
CM + 1.5%CR + 3.0%SR		5.90	1236	3.40	2.331	4.30	16.75	74.50	364
CM + 2.5%CR	3B	5.20	1136	3.61	2.268	4.30	15.20	72.00	315
CM + 2.5%CR + 1.5%SR		5.47	1191	3.86	2.273	3.67	15.20	76.00	308
CM + 2.5%CR + 2.0%SR		5.43	1282	3.63	2.260	4.40	15.65	73.00	353
CM + 2.5%CR + 2.5%SR		5.42	1209	3.89	2.275	3.80	15.10	75.00	311
CM + 2.5%CR + 3.0%SR		5.40	1141	3.61	2.265	4.20	15.45	73.00	316
CM + 5.0%CR		5.47	1109	3.91	2.201	4.20	15.30	72.50	283
CM + 5.0%CR + 1.5%SR		5.38	1159	3.79	2.203	4.20	15.15	72.50	306
CM + 5.0%CR + 2.0%SR		5.57	1182	3.81	2.200	4.10	15.45	73.50	310
CM + 5.0%CR + 2.5%SR		5.51	1127	3.91	2.347	4.30	15.40	72.00	288
CM + 5.0%CR + 3.0%SR		5.38	1095	3.94	2.206	4.00	15.00	73.00	278
Specification Limit [24]		(4–7)	≥ (1100)	(2–4)	------	(3–5)	≥ 14	≥ 70	------

### Direct compression (DC) and indirect tensile strength (ITS) test results

Table 9 summarizes the ITS and DC results for conditioned and unconditioned blends for all mixes. ITS was performed on asphalt mix samples prepared at the OAC, obtained by the Marshall test. The results indicated that adding CR and SR enhanced dry ITS values in both gradations. In dry conditions, the highest ITS enhancement occurred at (1 and 2.5) CR with 2% SR for gradations 4 C and 3B, respectively. The improvement was 70% and 59% for gradations 4 C and 3B, respectively. In wet conditions, WMA mixtures demonstrated greater ITS values across all SR percentages and gradations. The greatest improvement was at (1.0 and 2.5) % CR with 2%SR for gradations 4 C and 3B, respectively. Mixtures containing (1.0 and 2.5) % CR with 2%SR enhanced ITS (wet conditions) by 81% and 76% for gradations 4 C and 3B, respectively.

The DC test evaluates how materials behave and deform under static crushing stresses. The specimen is compressed with varying stress. The first specimen set is for the control mix without additives, whereas the other specimens were for the modified mixes with various percentages of SR and CR for the two gradations. DC results showed that adding CR and SR raised the dry DC values in both gradations. In dry conditions, the largest increase of DC occurred at (1 and 2.5) % CR with 2% SR for gradations 4 C and 3B, respectively. The increase was 34% and 40%. In wet conditions, WMA mixtures showed higher DC values across all SR percentages and gradations. The greatest improvement was at (1.0 and 2.5) % CR with 2%SR for grades 4 C and 3B, respectively.

**Table 9:** ITS and DC Values for Conditioned and Unconditioned Mixes Modified by CR and SR.

Mix Condition	Mix Gradation	ITS (Dry) ( kPa)	ITS (Wet) (kPa)	DC (Dry) (kPa)	DC (Wet) ( kPa)
CM (without additives)	4 C	795.35	645.30	2367.41	1669.15
CM + 0.5%CR		1009.14	849.29	2535.11	1901.58
CM + 0.5%CR + 1.5%SR		1098.38	991.49	2797.94	2152.64
CM + 0.5%CR + 2.0%SR		1239.60	1133.69	2947.98	2286.99
CM + 0.5%CR + 2.5%SR		1076.81	932.65	2758.71	2084.97
CM + 0.5%CR + 3.0%SR		1051.31	880.67	2592.97	1884.91
CM + 1.0% CR		1134.67	936.57	2574.34	1975.13
CM + 1.0%CR + 1.5%SR		1239.60	1053.27	2806.76	2162.44
CM + 1.0%CR + 2.0%SR		1349.44	1166.05	3174.53	2548.84
CM + 1.0%CR + 2.5%SR		1208.22	1029.74	2842.07	2166.37
CM + 1.0%CR + 3.0%SR		1105.25	910.09	2708.69	1967.28
CM + 1.5%CR		1066.02	881.65	2509.611	1802.527
CM + 1.5%CR + 1.5%SR		1134.67	998.35	2641.025	1963.361
CM + 1.5%CR + 2.0%SR		1167.03	1038.56	2877.374	2225.208
CM + 1.5%CR + 2.5%SR		1092.50	946.38	2795.976	2063.393
CM + 1.5%CR + 3.0%SR		1073.87	886.55	2738.114	2008.474
CM (without additives)	3B	678.64	551.15	1915.31	1352.39
CM + 2.5%CR		839.48	699.24	2219.32	1593.64
CM + 2.5%CR + 1.5%SR		896.36	766.91	2361.53	1848.62
CM + 2.5%CR + 2.0%SR		1079.75	967.95	2688.10	2158.52
CM + 2.5%CR + 2.5%SR		1023.85	880.67	2645.93	2069.28
CM + 2.5%CR + 3.0%SR		980.70	812.02	2569.43	2003.57
CM + 5.0%CR		708.07	594.30	2044.76	1526.95
CM + 5.0%CR + 1.5%SR		836.54	712.97	2167.35	1686.80
CM + 5.0%CR + 2.0%SR		947.36	875.77	2396.83	1936.88
CM + 5.0%CR + 2.5%SR		873.80	770.83	2365.45	1848.62
CM + 5.0%CR + 3.0%SR		751.22	626.67	2352.70	1720.15

### Wheel tracking test results

The wheel tracking test was carried out on asphalt mixes 0.5%,1%, and 1.5% of CR for gradation 4 C and 2.5% and 5% CR for gradation 3B, with the optimal proportion of SR (2%) for both gradations compared to the control mix (CM). Table 10 depicts rut depth values for HMA and WMA mixtures. It displays the ultimate rut depths for all mixtures at various CR and SR values. The results revealed an improvement when CR and SR were added to asphalt mixtures. The maximum improvement of rut depth for the modified asphalt mixes for gradations 4 C and 3B was at 1% and 2.5% CR and 2% SR, respectively. At this number, the rut depth fell by 44% and 40% at gradations of 4 C and 3B, respectively. Throughout the testing period, the rut depths of modified mixes were generally lower than those of control mixes (CM). The dynamic stability (represented by the slope of the linear line at the end of the rutting curve) was determined based on displacements at 45 and 60 min, mostly indicating the creep characteristics of the mixture during the shear deformation stage^33^. Finally, dynamic stability (DS) was calculated to determine the sample’s rutting deformation resistance. The dynamic stability (DS) was determined by Eq. (5).

5 $$\:\mathrm{D}\mathrm{S}\:=\:\frac{N\mathrm{*}\:15\:}{{d}_{60\:}-{\:d}_{45\:}}=\frac{42\mathrm{*}\:15\:}{{d}_{60\:}-{\:d}_{45\:}}$$

**Fig. 4:**
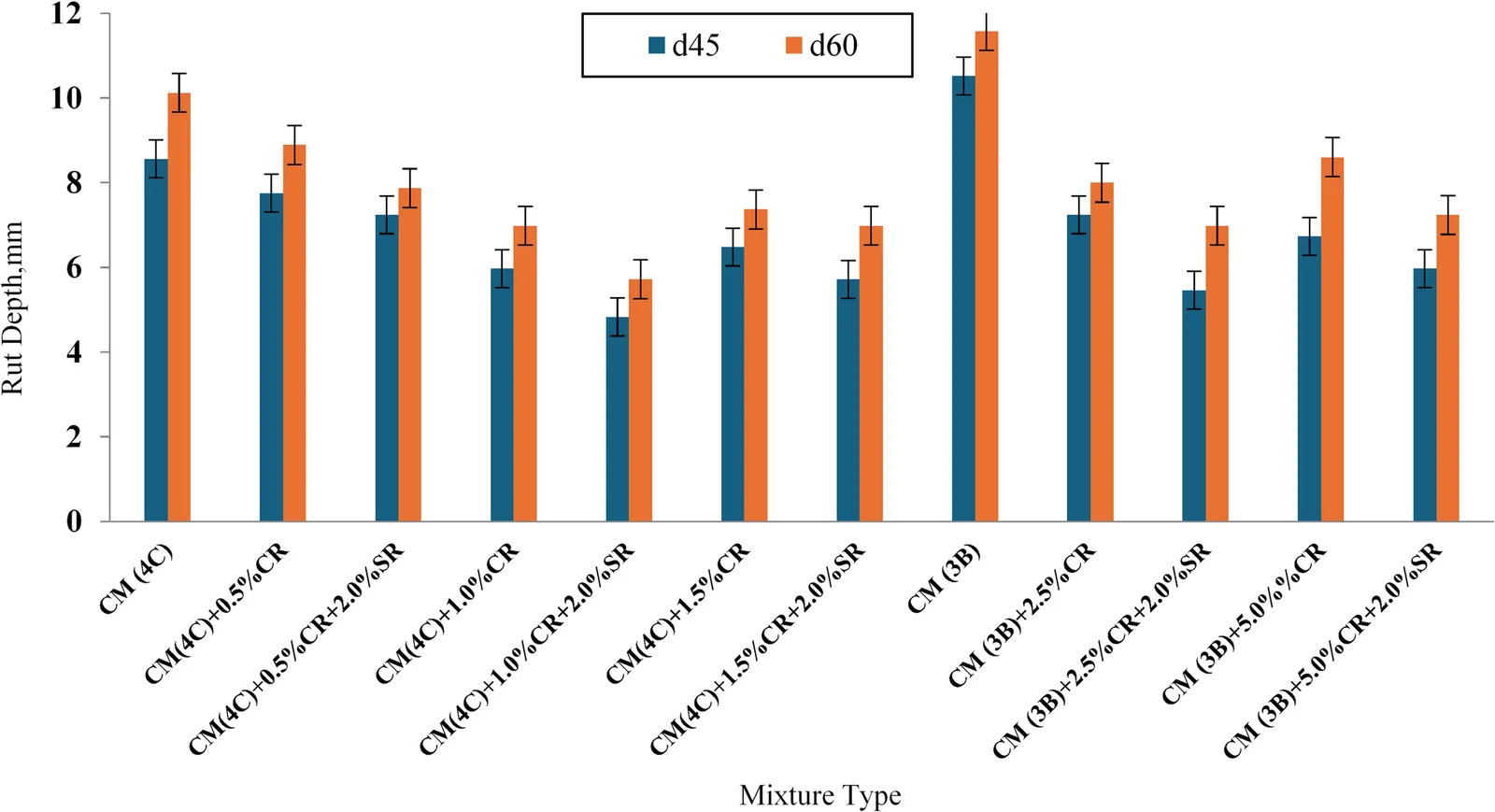
Rut depth values at 45 min (d_45_) and 60 min (d_60_).

**Fig. 5:**
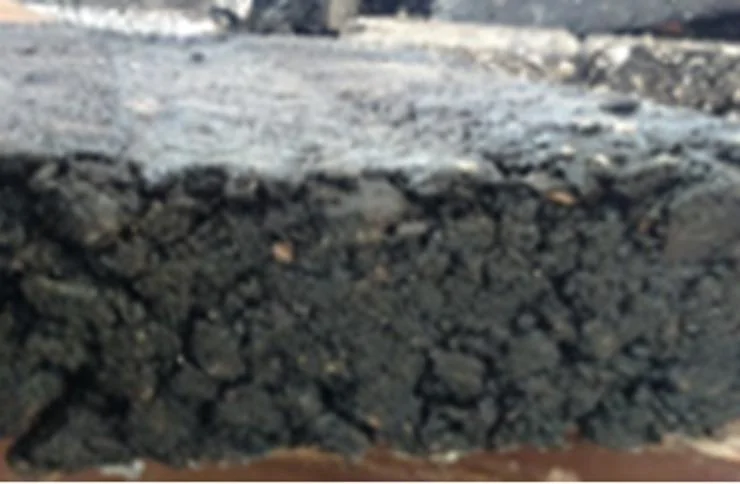
Shape of the specimen after test.

Where N is the number of wheel loading cycles per minute (42 in this case), and d_45_ and d_60_ are the rutting depths recorded at 45 and 60 min, respectively. For gradation 4 C, the modified mix (1% CR with 2% SR) had a lower rut depth and a greater DS than the control mix. In contrast, in gradation 3B, the modified mix (2.5% CR with 2% SR) had a lower rut depth and DS than the control mix. Table 10 presents the DS values, while Figure 4 shows the values of rut depth at 45 and 60 min. The results indicated that adding SR and CR enhanced the rut resistance in the 4 C mix more than in 3B. Figure 5 shows the shape of the specimen after the test.

**Table 10:** Results of the dynamic stability (DS) of wheel track test.

Asphalt Mixtures	DS (cycle/mm)
CM(4 C)	402
CM(4 C) + (0.5%CR)	551
CM(4 C) + (0.5%CR + 2.0%SR)	992
CM(4 C) + (1.0%CR)	620
CM(4 C) + (1.0%CR + 2.0%SR)	709
CM(4 C) + (1.5%CR)	733
CM(4 C) + (1.5%CR + 2.0%SR)	496
CM(3B)	594
CM(3B) + (2.5%CR)	827
CM(3B) + (2.5%CR + 2.0%SR)	413
CM(3B) + (5.0%CR)	331
CM(3B) + (5.0%CR + 2.0%SR)	496

### Triaxial shear test results

**Fig. 6:**
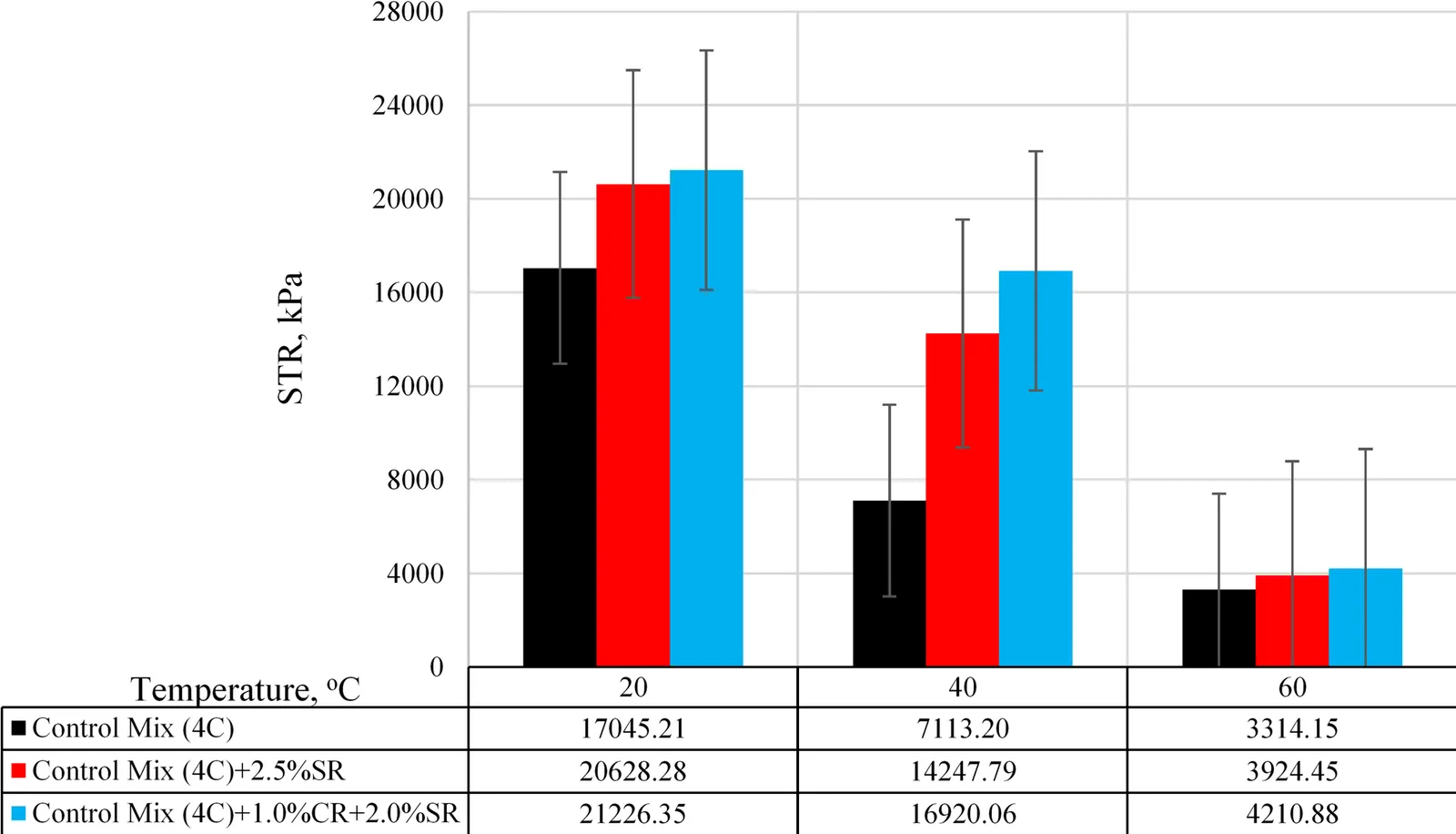
STR values for asphalt mixes for gradation 4 C.

**Fig. 7:**
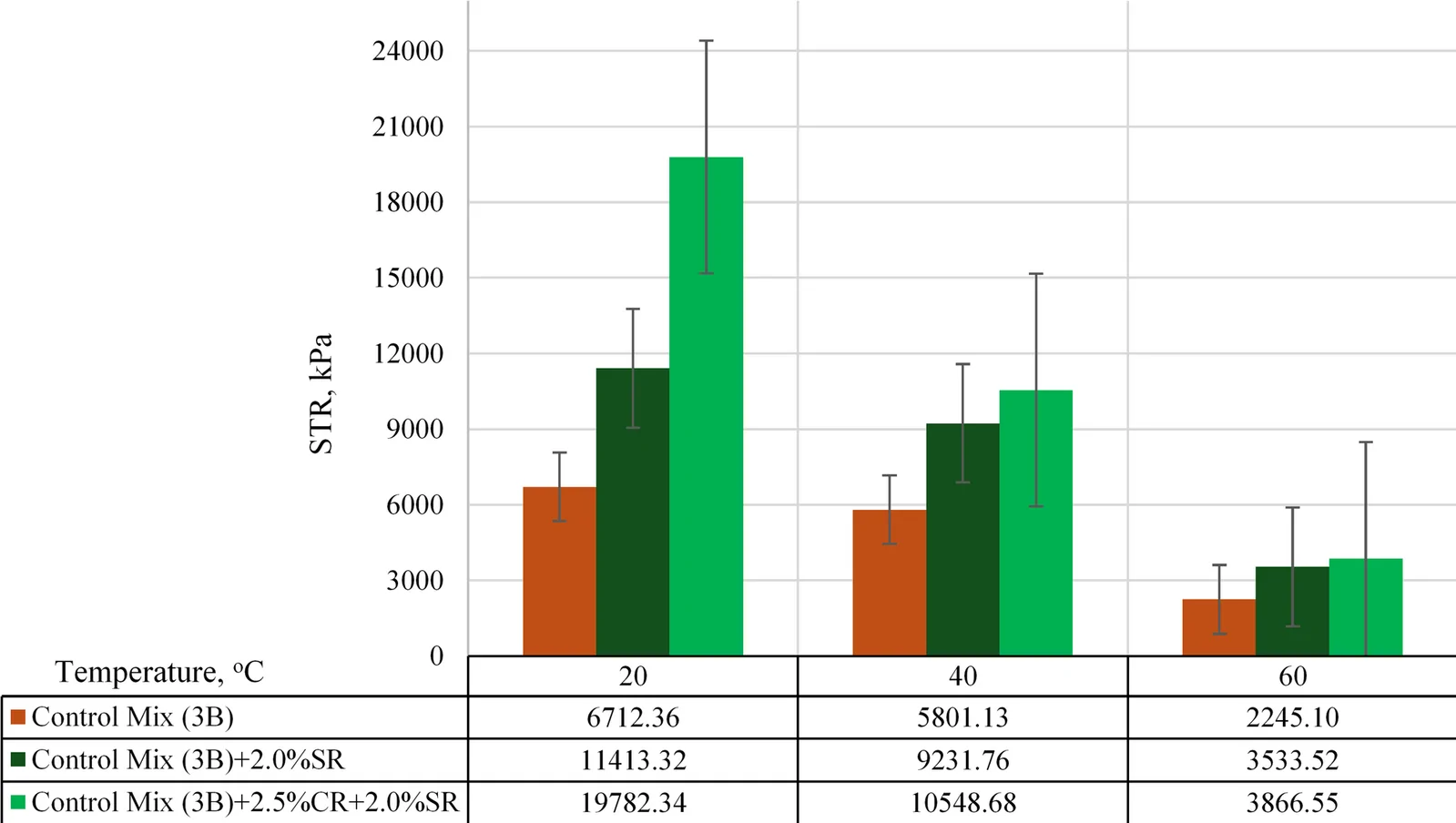
STR values for asphalt mixes for gradation 3B.

The results of the triaxial shear test (compressive strength versus lateral confining pressure) at different testing temperatures are presented in Table 11 for 4 C and 3B gradations. The values of cohesion (C) and angle of friction (Ф) were determined using the Mohr diagram. Then the corresponding values of shear triaxial resistance (STR) were calculated using Eq. (4). The effect of main variables related to testing conditions and mix compositions on the shear strength parameters (C and Ф) and shear triaxial resistance (STR) of asphalt concrete mixes has been investigated. These values of cohesion and friction angles were used in calculating the corresponding values of STR. The results showed enhancement in STR values at the optimum percentages for the two gradations (1.0% CR + 2% SR for gradation 4 C), (2.5% CR + 2% SR for gradation 3B). Figures 6 and 7 show the STR values for asphalt mixes for gradations 4 C and 3B, respectively. The variance in error bars is a well-documented physical trait of modified asphalt mixes in some cases and not a testing error.

**Table 11:** Triaxial Shear Test Results at Different Temperatures.

Temperature (℃)	20			40			60		
Mix Type	C (kPa)	Ф (^o^)	STR (kPa)	C (kPa)	Ф (^o^)	STR (kPa)	C (kPa)	Ф (^o^)	STR (kPa)
Control Mix (4 C)	440	47.35	17045.21	200	46.12	7113.20	120	42.27	3314.15
Control Mix (3B)	640	24.22	6712.36	160	46.40	5801.13	100	38.87	2245.10
Control Mix (4 C) + 2.5%SR	580	46.12	20628.28	260	52.00	14247.79	180	38.37	3924.45
Control Mix (4 C) + 1.0%CR + 2.0%SR	800	41.63	21226.35	400	48.58	16920.06	260	33.02	4210.88
Control Mix (3B) + 2.0%SR	680	33.69	11413.32	280	45.00	9231.76	140	40.82	3533.52
Control Mix (3B) + 2.5%CR + 2.0%SR	600	45.00	19782.34	310	45.47	10548.68	170	39.09	3866.55

### Dynamic modulus │E*│and flow number (FN) test results

**Fig. 8:**
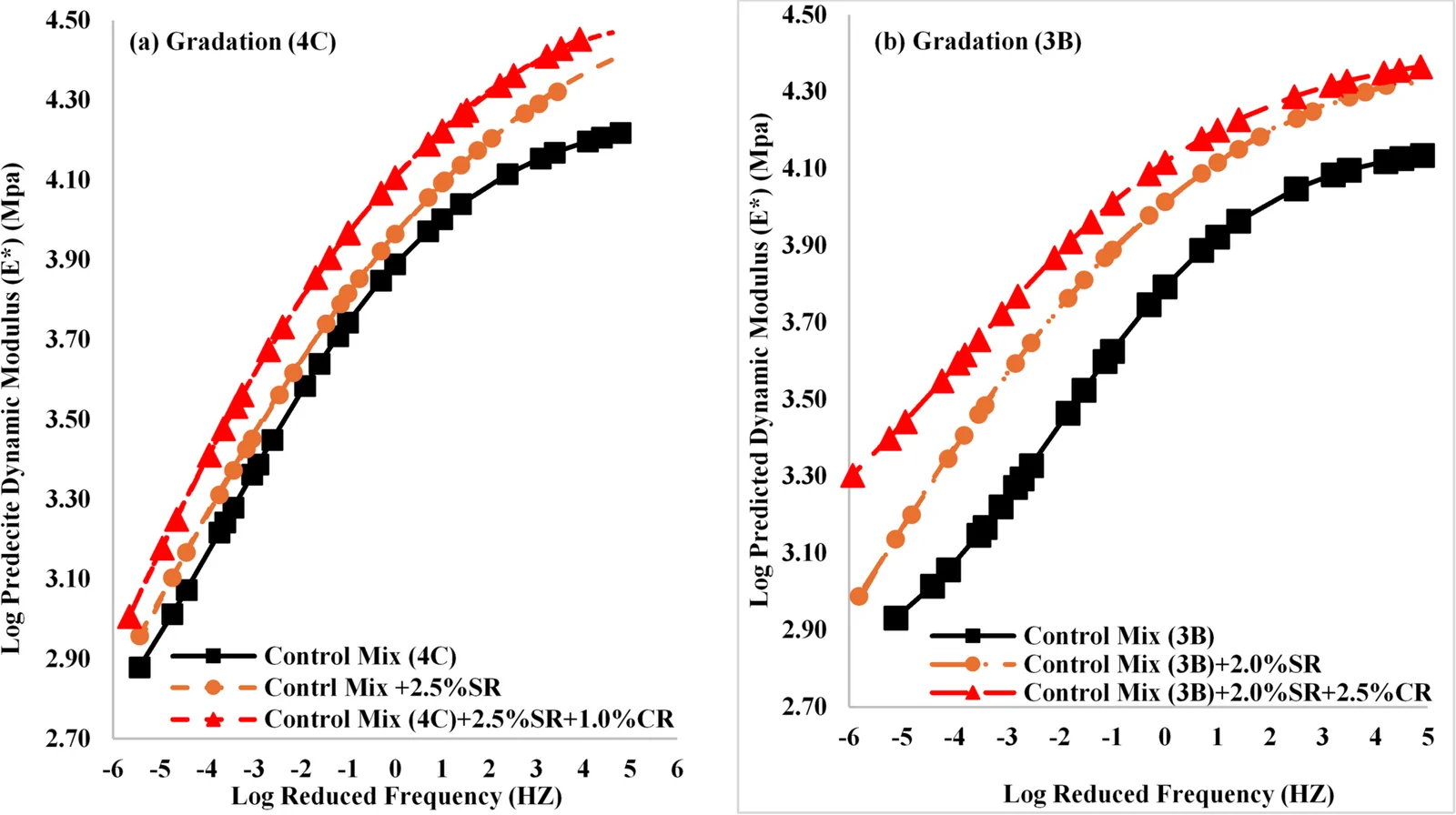
Master curve chart at different frequencies and different temperatures for different mixtures and gradations.

A dynamic modulus test was conducted for both control and modified mixtures. The findings demonstrated that the dynamic modulus │E*│decreased when temperature values increased at a specific frequency. Conversely, it was determined that mixtures including SR and CR exhibit superior performance compared to the control mixture in terms of dynamic modulus. The incorporation of SR with CR markedly increased the │E*│values in comparison to the control mixture. The results indicated that incorporating SR and CR into the control mix enhanced the performance of the modified mixes in resisting rutting across various temperatures and frequencies. The results were consistent with those derived by wheel tracking assessments. Master curves for each mixture were constructed by adjusting values relative to the reference temperature of 21.1 °C (70 °F) as per AASHTO-PP62 (2013). Figure 8 illustrates the Master curve for all mixtures. The figure presents master curves for control mixtures with varying gradations, modified with SR and CR, displayed on a log-log scale.

**Fig. 9:**
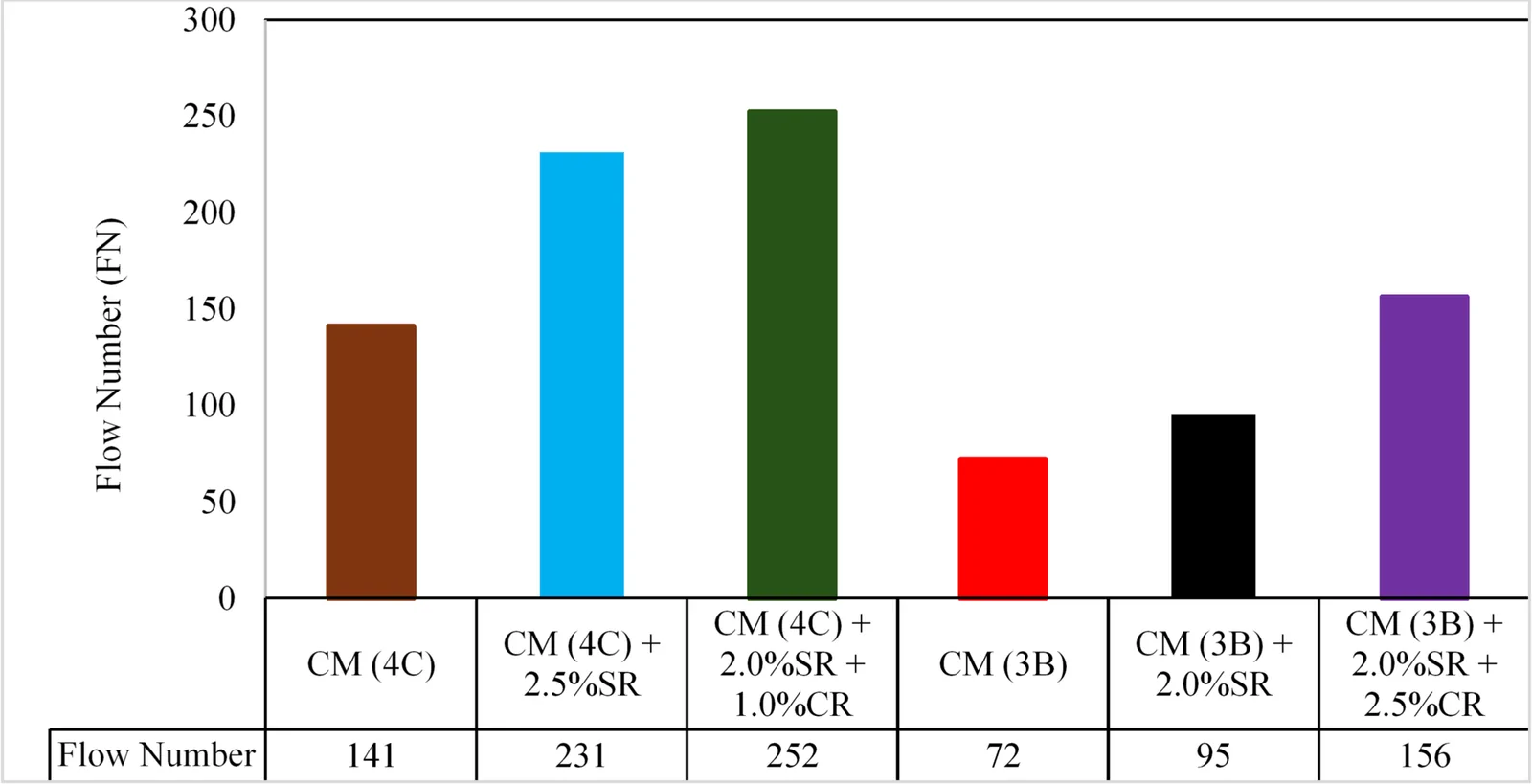
Flow number for different mixtures.

Figure 9 shows the comparison of the values of flow number (FN) for all mixtures. The results indicated that the mixture that contained 1.0% CR with 2.0% SR for gradation (4 C) and 2.5%CR with 2.0% SR for gradation (3B) had the maximum flow number (FN) compared to other mixtures. The FN test was a direct indication of rut resistance. These results confirm the wheel tracking test values, which consider the optimum percentage of 1.0% CR with 2.0% SR for gradation (4 C) and 2.5%CR with 2.0% SR for gradation (3B).

**Fig. 10:**
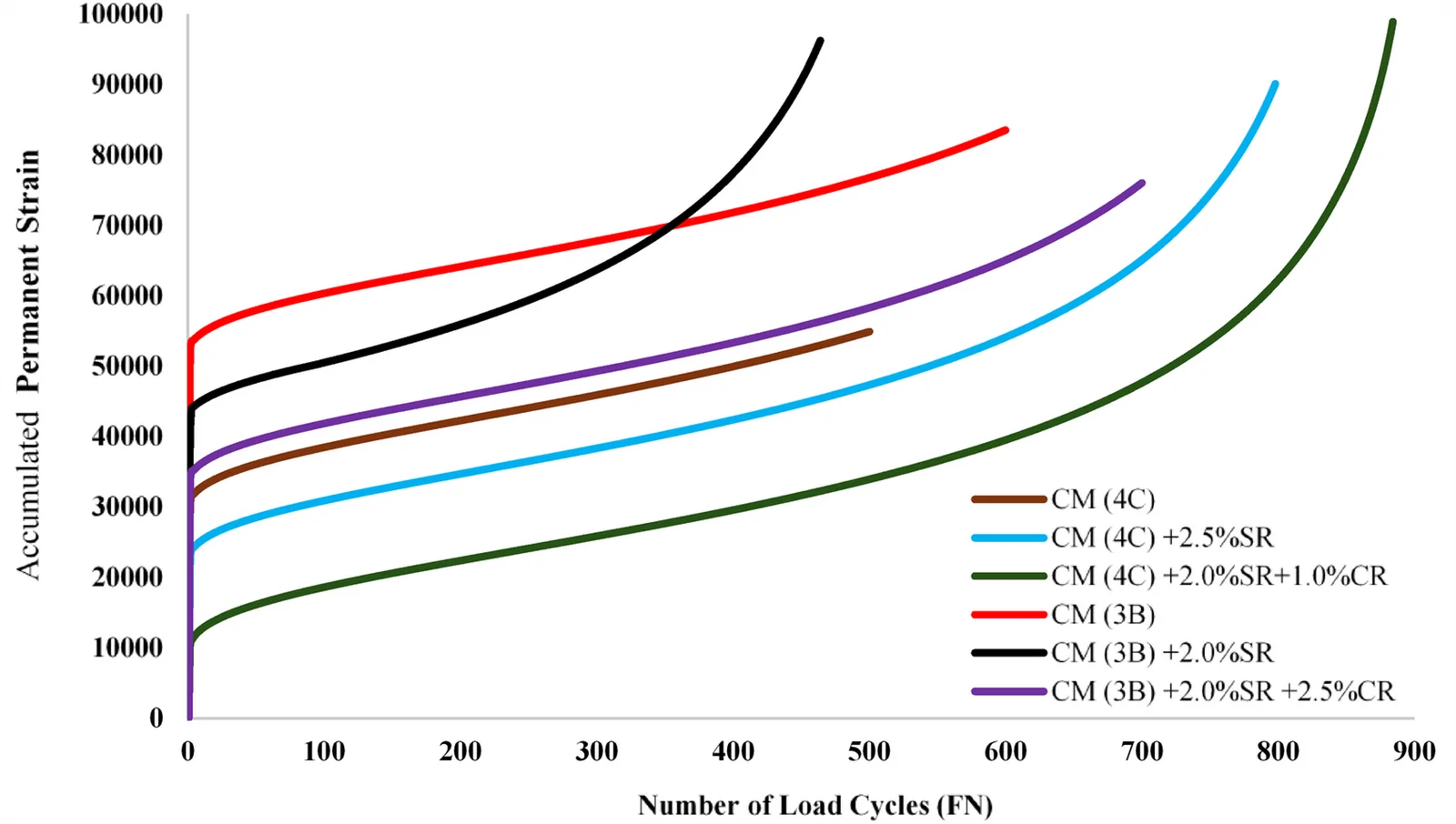
Accumulated permanent strain vs. number of load cycles (FN) for all mixes.

As noted previously, the flow number test was set to terminate at 100,000 micro-strains or set to terminate at 20,000 loading cycles. Thus, the accumulated permanent strain values versus several loading cycles for different mixtures are shown in Figure 10. This Figure shows accumulated permanent strain against the number of loading cycles. The results showed that mixtures containing 1.0% CR with 2.0% SR for gradation (4 C) and 2.5%CR with 2.0% SR for gradation (3B) introduced the best performance against rutting compared to the control and other mixes.

## Correlation analysis between asphalt mixture parameters

### Shear triaxial resistance (STR) prediction from the results of the common tests

**Fig. 11:**
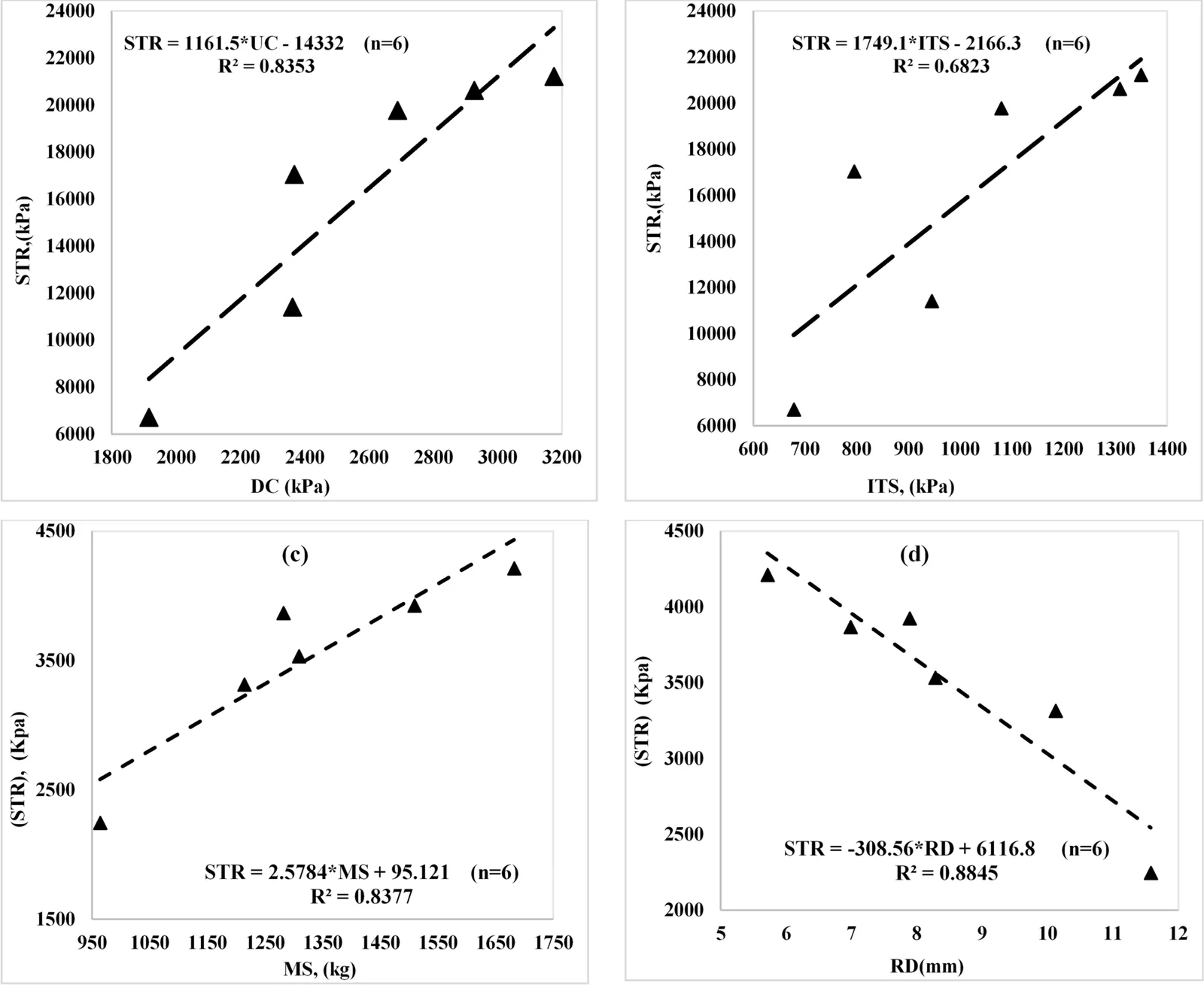
Correlation between shear triaxial resistance (STR) and (DC, ITS, MS, and RD) parameters.

Different techniques have been tried, aiming to establish a relationship to simplify the method of obtaining the STR of paving mixes and different parameters. These techniques are discussed in the following subsections. The method uses DC, ITS, MS, and RD to predict the relation of STR. The values of STR versus each of the investigated strength parameters were displayed as scatter diagrams to assess the acceptability of correlation. The calculated values of STR versus different parameters can be drawn and shown in Figure 11. The outcomes demonstrated that the relationship between STR and different parameters was proportional. Therefore, it can be deduced that there is a significant correlation between STR and the various parameters analyzed. All relations explained the high values of R^2^. It can be concluded that enhancing the engineering properties of asphalt mixes, such as DC, MS, ITS, and RD, enhanced the values of STR.

### Flow number (FN) prediction from rut depth (RD) at the simulative conditions in service

**Fig. 12:**
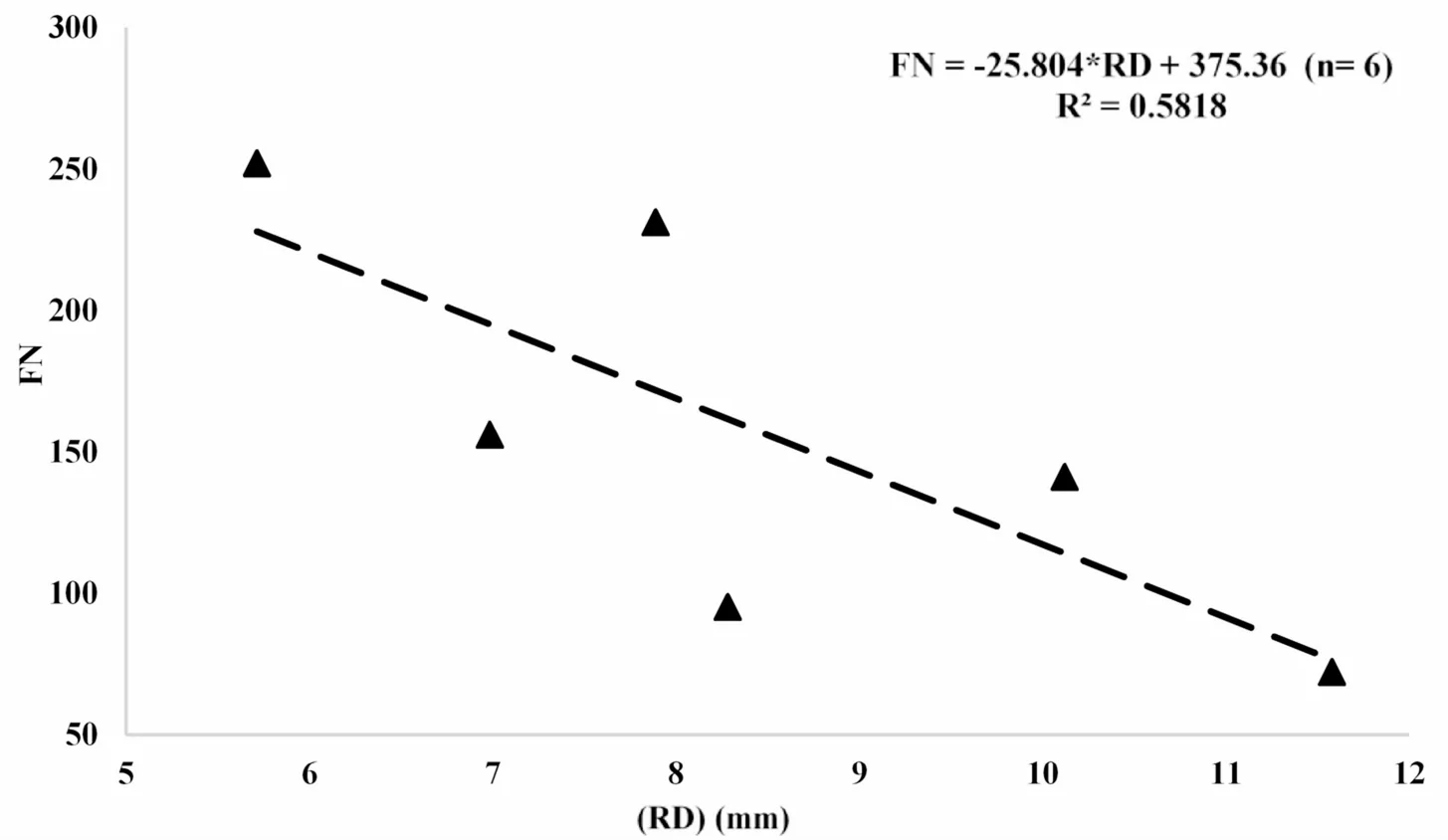
Correlation between Flow Numbers (FN) and Rut Depth (RD).

In this study, the correlation between FN and RD values was examined as shown in Figure 12. The figure shows that the relationship between FN and RD was inversely proportional. The correlation was conducted using six mixes, and it showed that FN decreased with the increase in RD values, with R^2^ = 0.5818.

### Dynamic modulus │E*│prediction from the results of the traditional tests

**Fig. 13:**
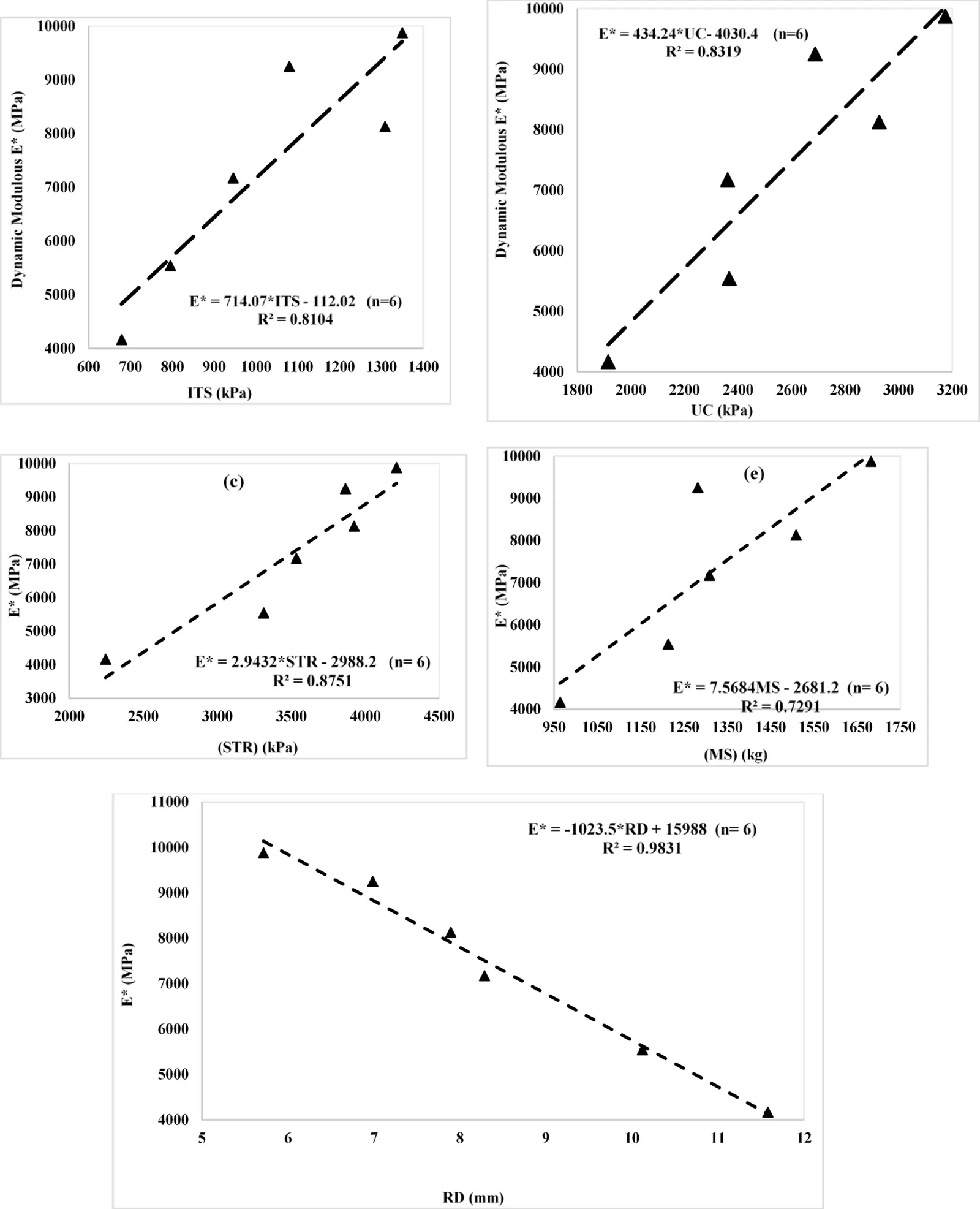
Correlation between Dynamic Modulus (E*) and (ITS, DC, STR, MS, and RD) Parameters.

Developing a guide criterion for paving mix stability under the imposed loads is not an effortless task. It must take into consideration all critical and practical conditions that influence factors around paving mixes in service. It was assumed that environmental and traffic conditions were the most effective factors affecting the performance of paving mixes in service. Environmental conditions were expressed here by temperature. The traffic conditions were considered here by frequency, which simulates traffic intensity. As demonstrated in the previous section, increasing temperature and frequency significantly affect the │E*│ of paving mixtures. The minimum value of │E*│was achieved in this study at a temperature of 54.4 °C, under other conditions. Furthermore, it was verified that the │E*│value at a temperature of 54.4 °C is about (1/5) the value of E* at 4.4 °C. Measuring dynamic modulus directly is not possible in most laboratories. So, reducing study data to mathematical form serves several purposes. Finding equations that correlate between │E*│ and routine mechanical properties could permit these laboratories to estimate the developed guide stability criterion. Therefore, different techniques have been tried, aiming to make correlations to simplify the method of obtaining the│E*│of paving mixes and different parameters. The suggested method is that of using indirect tensile strength (ITS) along with unconfined direct compression (DC), shear triaxial resistance (STR), Marshall Stability (MS), and rut depth (RD). The values of │E*│versus each one of the investigated strength parameters were displayed as scatter diagrams to assess the acceptability of correlation. The calculated values of │E*│ versus different parameters can be drawn and shown in Figure 13. The figures show that the relationship between │E*│ and different parameters is proportional. From these figures can deduce the equation between E* and different parameters can be deduced. A significant observation can be drawn from this analysis of the correlation of E* versus different parameters. This may be explained by the fact that the E* of paving mixes increased with the increase of different parameters. The values of │E*│used in the predictive correlations are carried out at a temperature of 37.78 °C, and a frequency of 25HZ.

### Applicability conditions and limitations of the developed correlations

It is essential to clarify the boundary conditions and scope of the developed regression models. The predictive correlations derived in this study are empirical and material-specific. The experimental dataset is strictly limited to one aggregate source, one binder grade, two specific gradations (4 C and 3B), and selected Sasobit redux (SR) and Crumb rubber (CR) contents under explicit laboratory testing conditions. The summary of laboratory test specifications and operating conditions for asphalt mixtures used in predictive correlations is presented in Table 12. Therefore, these correlations are valid only within the scope of these specific materials and variables. The regression equations should not be generalized to alternative asphalt binders, different aggregate types, varied CR gradations, other warm mix asphalt (WMA) additives, or diverse field conditions without rigorous independent validation.

**Table 12:** Summary of laboratory test specifications and operating conditions for asphalt mixtures used in predictive correlations.

Test	Specimen Dimensions		Rate of loading	Temperature (°C)	Specifications
	Diameter (Inch)	Height (inch)			
Marshall	4	2.5	50 mm/min	60	ASTM D 6927
Indirect Tensile Strength	4	2.5	50 mm/min	25	ASTM D6931
Unconfined Compression	4	4	5 mm/min	25	ASTM D1074
Wheel Tracking	305 mm×305 mm×50 mm			42 passes/ min	Egyptain Specifications & ASTM D8292
Triaxial Shear	4	4	50 mm/min	60	ASTM D2850
Dynamic Modulus	4	6	25 HZ	37.78	AASHTO-T312
Flow Number	4	6	0.1 and 0.9 s	54.4	AASHTO TP 79

### Statistical analysis of the predicted relations of dynamic modulus (E*), shear triaxial resistance (STR), and flow number (FN)

Statistical analysis using the multiple regression program (SPSS) was conducted between the predicted relations of │E*│, TSR, and FN and each one of the parameters investigated, separately, to differentiate between a general trend and the desired criterion from the previous parameters. Before a detailed evaluation of the correlation between the stated strength parameters of paving mixes and (│E*│, TSR, FN), it was necessary to determine the suitable form of the equation to be used. Statistical inferences (R^2^, Se, and F) were computed for different relations, using different equation forms. In statistical analysis, regression analysis is fundamentally important for quantifying relationships, predictive modeling, optimizing designs, and validating significance. A negligible improvement in the correlation coefficients was noticed by using higher-order equations rather than the linear form. This improvement was not of sufficient magnitude to warrant using the higher-order equation. So, only a linear relationship was adopted for all subsequent analyses. The results of the statistical analyses conducted revealed statistical inferences (R^2^, Se, and F), which are shown in Table 13. Statistical tables yielded an F (significance) value of α = 0.05 for a degree of freedom of 1 and residual N-2 = 4 (N = number of observations = 6).

**Table 13:** Statistical analysis parameters investigated from different relations using the SPSS program.

Regression Statistics	│E*│					STR				FN
	ITS	UC	STR	MS	RD	ITS	DC	MS	RD	RD
Multiple R	0.900	0.912	0.936	0.854	0.992	0.826	0.914	0.9152	0.940	0.763
R Square	0.810	0.832	0.875	0.729	0.983	0.682	0.835	0.8377	0.884	0.582
Adjusted R Square	0.763	0.790	0.844	0.661	0.979	0.603	0.794	0.7971	0.856	0.477
Standard Error	1067.50	1005.147	866.48	1276.046	318.645	3688.56	2655.65	313.9582	264.863	51.959
Observations	6									

The statistical studies yielded statistical conclusions (SS, MS, and F), as shown in Table 14. To determine the presence of these correlations, the multiple regression software (SPSS) was used to compute the F value. The value of F was higher than that of (F significance) for all data. This indicated that the relation between │E*│ and STR can be considered the most significant factor among all parameters investigated that can be used as an indicator of the paving mixes with a high degree of reliability.

**Table 14:** Statistical analysis parameters investigated from the different relations using the ANOVA test.

Dependent Variables	Independent Variables	ANOVA Test	df	SS	MS	F	Significance F
│E*│	ITS	Regression	1	19485064.97	19485064.97	17.10	0.0144
		Residual	4	4558226.74	1139556.68	------------	-------------
		Total	5	24043291.71	---------------	------------	-------------
	DC	Regression	1	20002007.83	20002007.83	19.80	0.0113
		Residual	4	4041283.87	1010320.97	------------	-------------
		Total	5	24043291.71	-------------	------------	-------------
	STR	Regression	1	21040138.45	21,040,138	28.02	0.0061
		Residual	4	3003153.26	750788.30	------------	-------------
		Total	5	24043291.71	-------------	------------	-------------
	MS	Regression	1	17530121.20	17530121.20	10.77	0.0305
		Residual	4	6513170.51	1628292.63	------------	-------------
		Total	5	24043291.71	-------------	------------	-------------
	RD	Regression	1	23,637,154	23637153.92	232.80	0.0001
		Residual	4	406137.80	101534.45	------------	------------
		Total	5	24,043,292	------------	------------	------------
STR	ITS	Regression	1	1.17E + 08	1.17E + 08	8.59	0.0428
		Residual	4	54,421,894	13,605,474	------------	------------
		Total	5	1.71E + 08	------------	------------	------------
	DC	Regression	1	143113528.27	143113528.27	20.29	0.0108
		Residual	4	28209916.69	7052479.17	------------	------------
		Total	5	171323444.96	------------	------------	------------
	MS	Regression	1	2034540.79	2034540.79	20.64	0.0105
		Residual	4	394278.92	98569.73	------------	------------
		Total	5	2428819.71	------------	------------	------------
	RD	Regression	1	2,148,209	2148209.24	30.62	0.0052
		Residual	4	280610.5	70152.62	------------	------------
		Total	5	2,428,820	------------	------------	------------
FN	RD	Regression	1	15023.85	15023.85	5.56	0.078
		Residual	4	10798.99	2699.75	------------	------------
		Total	5	25822.83	------------	------------	------------

Considering the conclusions deduced from the statistical analysis of data of the different parameters of the investigated mixes and │E*│, based on the results of laboratory work, it can be concluded that different parameters can be successfully used to indicate the │E*│of paving mixes. Whereas the │E*│ holds the potential for this purpose because of the relatively higher values of R^2^ associated with it. This means that STR can be recommended as an effective measure for ranking paving mixes according to their │E*│ with a high degree of reliability and correlation with them. Examining the correlation between │E*│ and other parameters, a reasonable correlation for │E*│with MS (R^2^ = 0.729, Se = 1276.05) and a strong correlation with the other parameters. Examining the correlation for │E*│ with STR reveals the value of R-squared (R^2^ = 0.875) with the value of the standard error (Se = 866.48). The correlation for │E*│ with RD reveals the highest value of R-squared (R^2^ = 0.983) with the smallest value of the standard error (Se = 318.64).

This finding suggests that STR and RD can be successfully used to reflect the │E*│ potential of paving mixes more than other parameters. However, it can be predicted that only about 83.20% of the │E*│ can be assessed by the DC. Whereas more than 87.50% of the │E*│ can be explained by the STR parameter. Whereas more than 98.31% of the │E*│ can be explained by the RD parameter. This denotes that improving the STR and RD of paving mixes would successfully minimize permanent surface deformation. The values of F for the conducted statistical analysis between │E*│and different considered parameters were also computed using the multiple regression program (SPSS). The F values for different correlations are also shown in Table 15. In contrast, based on the findings from laboratory work and the conclusions drawn from the statistical analysis of the various characteristics of the studied mixes and STR, we can conclude that MS and RD may be effectively used to indicate the STR potential of paving mixes. MS and RD can use this because of the higher R^2^ and F values associated with the smaller Se values. This indicates that MS and RD can be suggested as a reliable and efficient metric for grading paving mixes based on their STR resistance.

**Table 15:** Statistical inferences for the relation between dynamic modulus │E*│and STR and the investigated parameters of paving mix strength.

Dependent Variables	Independent Variables	*R* ^2^	Se	F
│E*│	ITS	0.8104	1067.50	17.09
	DC	0.8319	1005.15	19.80
	STR	0.8751	866.48	28.02
	MS	0.7291	1276.05	10.77
	RD	0.9831	318.64	232.79
STR	UC	0.8353	2655.65	20.29
	ITS	0.6823	3688.56	8.59
	MS	0.8377	313.99	20.64
	RD	0.8845	264.86	30.62

The Statistical analysis confirms that conventional Marshall Stability (MS) and Unconfined Compressive Strength (UC) strongly govern the internal friction and shear capacity of the mixture, explaining up to 83.77% and 83.53% of the STR variance. This establishes a highly reliable empirical connection between standard empirical tests and fundamental stress-state thresholds. By establishing these high-correlation models with high ANOVA significance (*p* < 0.05 and calculated F-values exceeding critical thresholds), it enables practicing engineers to reliably estimate advanced mechanistic pavement inputs (│E*│and STR) necessary for MEPDG software directly from routine, accessible plant tests (Marshall, DC, and Wheel Tracking).

## Conclusions

The conclusions of this study can be summarized as follows:

1. Modification of the asphalt mixtures with Crumb Rubber (CR) and Sasobit Redux (SR) significantly enhanced all conventional and mechanical performance indicators, including Marshall characteristics, Indirect Tensile Strength (ITS), Unconfined Compressive Strength (UCS), and wheel-tracking rut depths. Based on a comprehensive optimization of volumetric and strength parameters, the optimum modifier dosages were determined to be **(**1% CR + 2% SR) for the finer 4 C gradation and (2.5% CR + 2% SR) for the coarser 3B gradation.
2. Integrating Crumb Rubber (CR) altered the optimum modifier balance, shifting the required Sasobit Redux (SR) content from 2.5% in the previous study down to 2.0% in the current study.
3. The incorporation of Sasobit Redux (SR) and Crumb Rubber (CR) increased the dynamic modulus│E*│compared to the control mix, indicating potential improvements in rutting resistance under the evaluated laboratory conditions.
4. Within the experimental constraints, the highest │E*│ and Flow Number (FN) values were observed at specific combinations: 2.5%SR/1.0%CR for gradation (4 C) and 2.0%SR / 2.5%CR for gradation (3B).
5. The results of the Flow Number test confirmed the rutting test results and also verified the optimum additive contents, which are (1%CR + 2% SR) for gradation 4 C, and (2.5%CR + 2% SR) for gradation 3B.
6. Triaxial shear resistance (TSR) significantly decreased across all evaluated asphalt mixtures as the testing temperature increased. However, aggregate gradation 4 C exhibited superior STR values compared to gradation 3B.
7. Optimized modifier percentages (2.0%SR with 1.0%CR for 4 C, and 2.0%SR with 2.5%CR for 3B) enhanced the laboratory shear resistance of the mixtures across different temperatures.
8. Preliminary empirical equations were proposed to estimate │E*│and STR from conventional laboratory tests, demonstrating strong correlation coefficients (R²) within the specific scope and dataset of this study. These values were 0.9831 for the │E*│versus rut depth (RD) relationship and 0.8751 for the │E*│versus STR.
9. Statistical analysis confirms that conventional Marshall Stability and direct compression strongly govern mixture internal friction, explaining over 83% of shear triaxial resistance variance. Consequently, these highly significant ANOVA models (*p* < 0.05) establish a reliable empirical-mechanistic bridge, enabling practicing engineers to accurately estimate advanced MEPDG design inputs (│E*│and STR) directly from routine conventional tests.

## Data Availability

All data generated or analysed during this study are included in this published article.
